# Endograft Conformability in Fenestrated Endovascular Aneurysm Repair for Complex Abdominal Aortic Aneurysms

**DOI:** 10.1177/1526602820936185

**Published:** 2020-06-22

**Authors:** Arne de Niet, Esmé J. Donselaar, Suzanne Holewijn, Ignace F. J. Tielliu, Jan Willem H. P. Lardenoije, Clark J. Zeebregts, Michel M. P. J. Reijnen

**Affiliations:** 1 Department of Surgery (Division Vascular Surgery), University Medical Center Groningen, University of Groningen, the Netherlands; 2 Department of Surgery, Rijnstate, Arnhem, the Netherlands; 3 Multi-Modality Medical Imaging Group, Tech Med Centre, University of Twente, Enschede, the Netherlands

**Keywords:** abdominal aortic aneurysm, anatomical change, aortic diameter, conformability, fenestrated endograft, renal artery, stent-graft, visceral artery tortuosity index

## Abstract

**Purpose:** To compare the impact of 2 commercially available custom-made fenestrated endografts on patient anatomy. **Materials and Methods:** The records of 234 patients who underwent fenestrated endovascular aneurysm repair for abdominal aortic aneurysm from March 2002 to July 2016 in 2 hospitals were screened to identify those who had pre- and postoperative computed tomography angiography assessments with a slice thickness of ≤2 mm. The search identified 145 patients for further analysis: 110 patients (mean age 72.4±7.1 years; 94 men) who had been treated with the Zenith Fenestrated (ZF) endograft and 35 patients (mean age 72.3±7.3 years; 30 men) treated with the Fenestrated Anaconda (FA) endograft. Measurements included aortic diameters at the level of the superior mesenteric artery (SMA) and renal arteries, target vessel angles, target vessel clock positions, and the target vessel tortuosity index. Variables were tested for inter- and intraobserver agreement. **Results:** There was a good agreement between observers in all tested variables. The native anatomy changed in both groups after endograft implantation. In the ZF group, changes were seen in the angles of the celiac artery (p=0.012), SMA (p=0.022), left renal artery (LRA) (p<0.001), and the right renal artery (RRA) (p<0.001); the aortic diameter at the SMA level (p<0.001); and the LRA (p<0.001) and RRA (p<0.001) clock positions. In the FA group, changes were seen in the angles of the LRA (p=0.001) and RRA (p<0.001) and in the SMA tortuosity index (p=0.044). Between group differences in changes were seen for the aortic diameters at the SMA and renal artery levels (p<0.001 for both) and the LRA clock position (p=0.019). **Conclusion:** Both custom-made fenestrated endografts altered vascular anatomy. The data suggest a higher conformability of the Fenestrated Anaconda endograft compared with the Zenith Fenestrated.

## Introduction

The treatment of choice for abdominal aortic aneurysm (AAA) repair has shifted from open repair toward endovascular aneurysm repair (EVAR), mainly because of favorable early results.^1–4^ The introduction of fenestrated endografts enabled endovascular treatment of short-necked, juxtarenal, and suprarenal AAAs. The fenestrations maintain flow through the visceral arteries, while the endograft is landed in a healthy neck above the aneurysm.^5^ Preoperative assessment of aortoiliac morphology is essential for optimal case planning. These measurements, which are typically performed using a central lumen line (CLL) to generate 3-dimensional (3D) reconstructions from computed tomography angiography (CTA) scans of the aorta and branch arteries, have good interobserver agreement for both standard and complex EVAR planning.^6–9^

The Cook Zenith Fenestrated endograft (Cook Medical Inc, Bloomington, IN, USA) was the first commercially available fenestrated design. It consists of self-expanding stainless steel Z-stents covered with full-thickness woven polyester fabric containing fenestrations between the struts of the Z-stents.^10,11^ The more recently introduced Fenestrated Anaconda endograft (Terumo Aortic, Inchinnan, Scotland, UK) has independent nitinol rings, a woven polyester graft, and an unsupported proximal body containing fenestrations.^12,13^ In both models, the fenestrations and target vessels are cannulated and stented after deployment of the main body. The choice of endograft is mostly based on the experience and preference of the clinician.

EVAR may change the native anatomy of the patient.^14^ Such a conformational change may lead to a proximal seal zone failure through infrarenal aortic angle change or iliac limb complications through changes in iliac artery tortuosity.^15,16^ Different infrarenal endograft designs have different conformability, so the choice of endograft influences the risk of complications.^16,17^ Endograft implantation and placement of stents in the target vessels influence arterial angle and curvature after fenestrated EVAR (fEVAR).^18^ Altered anatomy could potentially kink the stented target vessel, strain the endograft, lead to material fatigue, or create thrombosis and distal emboli.

The exact influence of fenestrated endograft implantation on human aortic anatomy is unknown.^19^ The differences in design of commercial fenestrated endografts may impose different local changes in anatomy, which might be of importance for graft-related complications, particularly involving the stents in the target vessels. The aim of the present study was to assess the conformability of the Zenith Fenestrated (ZF) and Fenestrated Anaconda (FA) endografts and to study the differences in anatomical changes after placement.

## Materials and Methods

### Study Design and Patient Sample

The records of 234 patients who underwent fEVAR for AAA (no thoracic implantations or branches) from March 2002 to July 2016 in 2 Dutch hospitals were screened to identify those who had pre- and postoperative CTA assessments with a slice thickness of ≤2 mm. The search identified 145 patients for further analysis after exclusion of 89 patients not meeting the imaging criterion. A total of 110 patients (mean age 72.4±7.1 years; 94 men) had been treated with the ZF endograft and 35 patients (mean age 72.3±7.3 years; 30 men) with the FA endograft. Baseline patient characteristics are shown in Table 1.

**Table 1.** Baseline Patient Characteristics by Type of Endograft.^a^

Variable	Zenith Fenestrated (n=110)	Fenestrated Anaconda (n=35)	p
Age, y	72.4±7.1	72.3±7.3	0.94
Men	94 (85.5)	30 (85.7)	0.97
BMI, kg/m^2^	27.6±3.9	28.7±4.5)	0.20
Plasma creatinine, μmol/L	89 (77, 109)	94 (76, 108)	0.88
Smoker	39 (35.5)	10 (28.6)	0.14
Previous	25 (22.3)	10 (28.6)	
Unknown	6 (5.5)	10 (28.6)	
Hypertension^b^	89 (80.9)	28 (80.0)	0.27
1	39	10	
2	34	8	
3	16	10	
Hypercholesterolemia	77 (70.0)	28 (80.0)	0.25
Diabetes mellitus	13 (11.8)	5 (14.3)	0.77
Stroke/TIA	14 (12.7)	3 (8.6)	0.36
Peripheral artery disease	9 (10.0)	5 (14.3)	0.53
Cardiac disease^b^	68 (61.8)	16 (45.7)	0.015
1	21	11	
2	32	5	
3	15	0	
Pulmonary disease^b^	38 (34.5)	4 (11.4)	0.028
1	17	4	
2	15	0	
3	6	0	
Previous operation			0.59
Open	10 (9.9)	1 (2.9)	
EVAR	8 (7.2)	3 (8.6)	
ASA class			0.19
II	29 (26.4)	15 (42.9)	
III	73 (66.4)	20 (57.1)	
IV	2 (1.8)	0	
Unknown	6 (5.5	0	
Aneurysm location	43 (39.1)	20 (57.1)	0.27
Infrarenal			
Juxtarenal	56 (50.9)	13 (37.1)	
Suprarenal	11 (1.8)	2 (5.7)	
Type IV TAAA	2 (1.8)	0	

Abbreviations: BMI, body mass index; COPD, chronic obstructive pulmonary disease; EVAR, endovascular aneurysm repair; TAAA, thoracoabdominal aortic aneurysm; TIA, transient ischemic attack.

a Continuous data are presented as the mean ± standard deviation or median (interquartile range Q1, Q3); categorical data are given as the number (percentage).

b As described by Chaikof et al.^21^

Preoperative patient characteristics were extracted from the charts, including the American Society of Anesthesiologists (ASA) class.^20^ Patient characteristics were classified according to the reporting standards of the Society of Vascular Surgery (SVS) and the SVS score related to perioperative mortality risk.^21^

The study was conducted in accord with the principles of the Declaration of Helsinki and Good Clinical Practice guidelines. Retrospective medical records research is not in the scope of the Dutch law governing research involving human beings, thus the Institutional Review Board issued a waiver (reference number M17.207929) so no informed consent was obtained. Patient data were anonymized.

### Analysis of CTA Parameters

**Figure 1.**
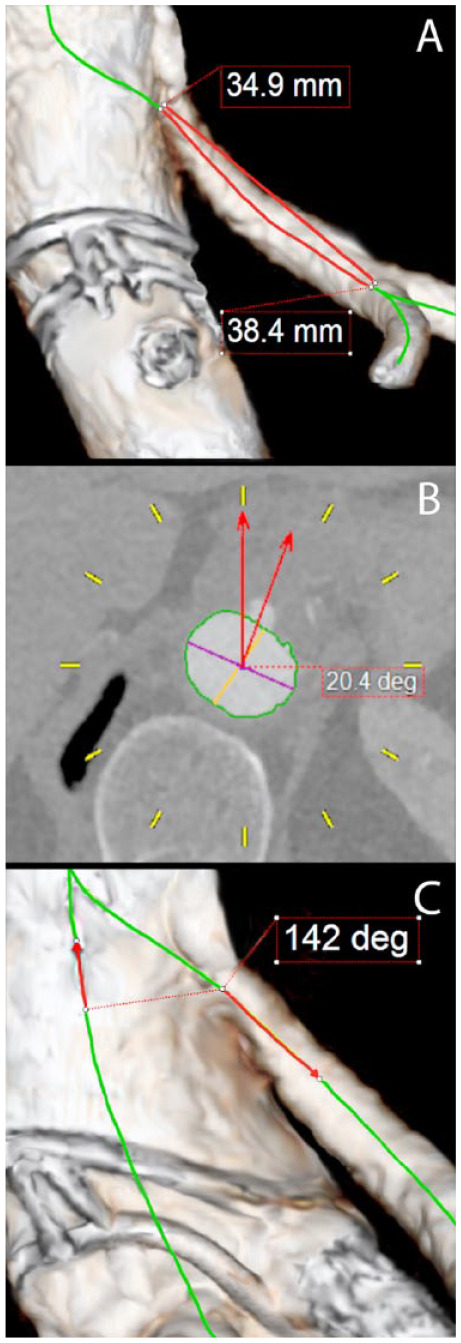
Reconstructions of the aorta from computed tomography angiography measurements. (A) Tortuosity index: measuring the distance over the central lumen line (CLL) from the origin of the target vessel to the first bifurcation of >50% in diameter of the main branch. (B) Clock position: the spine orientation is reset on the dorsal side and the target vessel is measured relative to a straight line from the CLL to 12 o’clock. Time after 12 is labeled positive (+) and before 12 is labeled negative (–). (C) Target vessel angle: measured from the vessel origin to 1 cm from the origin relative to the CLL of the aorta. Perpendicular orientation is 90° and downward orientation is toward 180°, while upward orientation is toward 0°.

Measurements using the pre- and first postoperative CTA scans were performed using Aquarius iNtuition (version 4.4.7; TeraRecon, Foster City, CA, USA) and Philips IntelliSpace Portal (version 8.0; Philips Healthcare, Eindhoven, the Netherlands). An automatically drawn CLL was manually adjusted when necessary, and 3D reconstructions were automatically created. Measurements included the maximum aortic diameter between the upper and lower margins of the superior mesenteric artery (SMA) and between the upper margin of the most cranial renal artery and lowest margin of the most caudal renal artery. All target vessels were measured for the tortuosity index (Figure 1A),^22^ clock position (Figure 1B), and angle relative to the aortic CLL (Figure 1C). On the postoperative CTA, 2 straight lines were drawn along 3 points on the CLL of the stented target vessel. The first point was placed at the distal marker of the target vessel stent, the second was placed 1 cm proximal to the distal marker, and the third was placed 1 cm distal of the distal marker. One straight line was drawn from point 1 to point 2 and the second straight line from point 1 to point 3. The angle between both straight lines was measured.

### Definitions and Statistical Analysis

Technical success was considered an endograft deployment as planned, including stented fenestrations, in the absence of type I or III endoleak, conversion, or death up to 24 hours postoperatively. Assisted technical success applied to cases in which an endovascular adjunctive procedure was necessary during the first 24 hours postoperatively.^23^

Continuous variables were tested for normal distribution by observation of Q-Q plots and reported in the tables. Normally distributed variables were reported as the mean ± standard deviation; variables with a skewed distribution were given as the median [interquartile range (IQR) Q1, Q3]. Discrete variables are presented as frequencies (percentage).

Differences in continuous data between groups of baseline patient and anatomical characteristics were tested with the Student *t* test or the Mann-Whitney *U* test for skewed data. Differences in discrete data between groups were tested with the Fisher exact test. Changes in anatomy within groups were tested with paired Student *t* test or the Wilcoxon signed-rank test for skewed data. The difference in anatomical change between balloon-expandable (BE) covered stents, self-expanding (SE) bare metal stents, or a combination within groups were tested with the analysis of variance for repeated measures or the Kruskal Wallis test for single measures.

To define intra- and interobserver variability, the first observer (A.N.) did all tested measurements in both systems and did repeated measurements of pre-/postoperative CTA scans in 20 randomly assigned cases using the Aquarius iNtuition workstation. The second observer (E.D.) measured variables from pre-/postoperative CTA scans in 20 randomly assigned cases using the Aquarius iNtuition software. The second observer measured variables (pre-/postoperative CTA) in 20 randomly assigned cases using the Philips IntelliSpace software and did repeated measurements in the same postoperative CTAs. Consistency agreement was tested with the 2-way mixed intraclass correlation coefficient (ICC). Based on the ICC, reliability was considered poor for values <0.500, moderate for values between 0.500 and 0.750, good for values between 0.750 and 0.900, and excellent over 0.900.^24^ Observer 1 repeatedly measured with Aquarius iNtuition and measured 20 randomly assigned cases with Philips IntelliSpace, and observer 2 repeatedly measured with Philips IntelliSpace and measured 20 randomly assigned cases with Aquarius iNtuition. Both operating systems were analyzed separately for observer variability. Methods of measuring the aortic diameter and visceral target arteries were identical within each operating system, so the data were combined to test observer variability. Combined variables were aortic diameters at the SMA and at the renal arteries, all tortuosity indices (both pre- and postoperative vessels), all clock positions, all target vessel angles relative to the aortic CLL, and all target vessel angles distal to the stent.

Analysis of anatomical change within and between groups was performed with separate variables and in stented target vessels only. P<0.05 was considered the threshold of statistical significance. Statistical analysis was done with IBM SPSS software (version 23.0.0.3; IBM Corporation, Armonk, NY, USA).

## Results

### Procedure Outcomes

Technical success was 88.2% (n=97) in the ZF group and 82.9% (n=29) in the FA group (p=0.402); assisted technical success was 91.8% (n=101) in the ZF group and 92.9% (n=29) in the FA group (p=0.198). In the ZF group, the target vessels were stented with BE covered stents in 85 cases (77.3%), SE bare metal stents in 20 cases (18.2%), and a combination in 5 cases (4.5%). The cases with SE bare metal stents were the first treated fEVAR cases. In the FA group, all target vessels were stented with a BE covered stent.

Table 2 shows details of the procedures for both groups. In the ZF group, 36 intraoperative adjunctive procedures were performed in 33 patients. Additional stenting of a target vessel was necessary in 12 cases because of kinking, stenosis, endoleak, an overly short stent, or a puncture in a target vessel. One patient had an embolus to the right kidney successfully treated with thrombolysis. In 4 cases the overlap between main endograft components was insufficient and an additional cuff was placed. The other adjunctive procedures were treatments to an iliac or femoral artery. A total of 11 adjunctive procedures were performed in 10 patients in the FA group. Additional angioplasty was done for nonstented visceral arteries in 2 cases and reinforcing stenting of a stented target vessel in 1 case. In 1 case there was an inability to cannulate the celiac artery (CA); a proximal extension cuff was introduced to seal the fenestration and prevent endoleak. All other adjunctive procedures concerned the iliac or femoral artery.

**Table 2.** Details of the Fenestrated Endograft Implantations.^a^

Variable	Zenith Fenestrated (n=110)	Fenestrated Anaconda (n=35)	p
Procedure time, min	215 (180, 291)	238 (170, 315)	0.81
Contrast volume, mL	180 (150, 220)	130 (116, 194)	0.001
Estimated blood loss, mL	250 (150, 497)	100 (63, 175)	<0.001
Iliac extension	96 (87.3)	31 (88.6)	0.40
Bifurcated	96 (87.3)	31 (88.6)	
Monoiliac	2 (1.8)	2 (5.7)	
Cuff	12 (10.9)	2 (5.7)	
Fenestrations	2.4±0.8	2.6±0.8	
1	16 (14.5)	2 (5.7)	0.49
2	49 (44.5)	15 (42.9)	
3	36 (32.7)	14 (40.0)	
4	9 (8.2)	4 (11.4)	
Adjunctive procedure	33 (30)	10 (28.6)	0.87
Endovascular	30 (27.3)	8 (22.9)	0.59
Open	6 (5.5)	3 (8.6)	0.69

a Continuous data are presented as the mean ± standard deviation or median (interquartile range Q1, Q3); categorical data are given as the number (percentage).

At completion angiography, there were more endoleaks in the ZF group (34, 31.0%) vs the FA group (18, 51.4%; p=0.027), including a type Ia endoleak in 7 ZF cases (6.4%) and in 6 FA cases (17.1%; p=0.053). On the postoperative CTA, 2 type Ia endoleaks (1.8%) were diagnosed in the ZF group vs 1 (2.9%) in the FA group (p=0.391). No reinterventions had been performed to resolve an endoleak until after the first postoperative CTA. Type II endoleaks were seen in 26 cases (23.6%) in the ZF group and 12 (34.3%) in the FA group (p=0.214). At the first postoperative visit, 1 type III endoleak was seen in the ZF group (0.9%) vs none in the FA group (p=0.573).

### Anatomical Change Analysis

Time between baseline CTA and treatment was 4.0 months (IQR 3.1, 5.1) in the ZF group and 3.3 months (IQR 2.3, 4.3) in the FA group. Time between treatment and first postoperative CTA was 1.4 months (IQR 1.1, 1.7) in the ZF group and 1.0 month (IQR 0.1, 1.8) in the FA group. Time between baseline CTA and treatment was shorter in the FA group (p=0.012), and no difference was seen between groups in the interval between the operation and the first postoperative CTA scan (p=0.055).

Table 3 shows the combined variables with the ICC values. All tested variables had moderate, good, or excellent intra-/interobserver reliability and therefore could be used for analysis in this study. Baseline differences in anatomy are shown in Table 4. Fifteen fenestrations were used for the CA (10 in the ZF group and 5 in the FA group) and 65 fenestrations for the SMA (47 in the ZF group and 18 in the FA group). The baseline aortic diameter at the level of the SMA was 2 mm larger in the ZF group compared with the FA group (p=0.007).

**Table 3.** Intra- and Interobserver Variability.^a^

Measurements	Intraobserver		Interobserver	
	Observer 1^b^	Observer 2^c^	iNtuition^d^	Intellispace^e^
Aortic diameter	80	80	80	80
Mean (diff), mm	25.9 (0.1)	27.4 (0.3)	26.4 (1.2)	26.2 (0.9)
ICC (95% CI)	0.946 (0.917 to 0.965)	0.930 (0.872 to 0.963)	0.747 (0.632 to 0.830)	0.761 (0.651 to 0.840)
Target vessel tortuosity index	160	77	160	154
Mean (diff)	1.11 (0)	1.11 (0)	1.13 (0.03)	1.11 (0.01)
ICC (95% CI)	0.725 (0.642 to 0.791)	0.931 (0.893 to 0.955)	0.818 (0.759 to 0.863)	0.822 (0.764 to 0.868)
Target vessel clock position	160	77	160	154
Mean (diff)	14.3 (1.3)	10.1 (0.3)	11.4 (4.4)	10.0 (0.8)
ICC (95% CI)	0.963 (0.950 to 0.973)	0.999 (0.998 to 0.999)	0.942 (0.921 to 0.957)	0.954 (0.937 to 0.966)
Target vessel angle	160	77	160	151
Mean (diff), deg	124.2 (0.2)	112.7 (0.5)	124.0 (0.7)	116.0 (0.1)
ICC (95% CI)	0.845 (0.794 to 0.884)	0.946 (0.916 to 0.965)	0.696 (0.607 to 0.768)	0.590 (0.475 to 0.685)
Target vessel angle distal of stent	50	52	50	52
Mean (diff), deg	153.8 (1.5)	144.9 (0.8)	154.1 (0.6)	151.0 (12.9)
ICC (95% CI)	0.750 (0.598 to 0.850)	0.924 (0.871 to 0.956)	0.717 (0.549 to 0.829)	0.561 (0.343 to 0.722)

Abbreviations: CI, confidence interval; diff, difference; ICC, intraclass correlation coefficient.

a The mean measurement is shown with the mean difference (diff) between measurements. Pre– and post–computed tomography angiography (CTA) data were combined; see the text for details.

b Repeated measurements by observer 1 were done in 20 randomly assigned cases (combined pre-/postoperative CTA) using Aquarius iNtuition.

c Measurements by observer 2 were done in 20 randomly assigned cases using Philips IntelliSpace and compared to repeated measurements in the postoperative CTA.

d Measurements by observer 2 (combined pre-/postoperative CTAs) of 20 randomly assigned cases in Aquarius iNtuition were compared to initial measurements in the same cases by observer 1.

e Measurements by observer 2 (combined pre-/postoperative CTAs) of 20 randomly assigned cases in Philips Intellispace were compared to initial measurements by observer 1 in the same cases.

**Table 4.** Preoperative Anatomy Measurements for Stented Target Vessels Only.^a^

Variable	Zenith Fenestrated	Fenestrated Anaconda	p
Aortic diameter at the SMA, mm	27 (25, 30) [110]	25 (23, 28) [35]	0.007
Aortic diameter at the RA, mm	27 (25, 33) [110]	28 (23, 31) [35]	0.16
CA tortuosity index	1.08 (1.05, 1.12) [10]	1.05 (1.04, 1.11) [5]	0.44
CA clock position, deg	14 (3, 25) [10]	21 (14, 25 [5]	0.59
Angle of the CA, deg	137 (124, 147) [10]	123 (121, 129) [5]	0.17
SMA tortuosity index	1.04 (1.02, 1.07) [47]	1.04 (1.02, 1.11) [18]	0.52
SMA clock position, deg	7 (−10, 14) [47]	7 (2, 14) [18]	0.54
Angle of the SMA, deg	127 (115, 136) [47]	120 (118, 127) [18]	0.56
LRA tortuosity index	1.10 (1.05, 1.18) [105]	1.15 (1.05, 1.24) [32]	0.21
LRA clock position, deg	84±18 [105]	86±20 [32]	0.72
Angle of the LRA, deg	114±17 [105]	117±15 [32]	0.46
RRA tortuosity index	1.12 (1.05, 1.19) [98]	1.15 (1.11, 1.22) [34]	0.02
RRA clock position, deg	−68±17 [98]	−64±15 [34]	0.15
Angle of the RRA, deg	117±16 [98]	117±19 [34]	0.63

Abbreviations: CA, celiac artery; LRA, left renal artery; RA, renal arteries; RRA, right renal artery; SMA, superior mesenteric artery.

a Continuous data are presented as the mean ± standard deviation or median (interquartile range Q1, Q3) [sample size]; categorical data are given as the number (percentage).

Table 5 shows changes within groups and differences in changes between groups for all variables from the preoperative and first postoperative CTA scans. A statistically significant change within the ZF group was seen for the aortic diameters at the level of the CA and SMA; the angles of the CA, SMA, LRA, and RRA; and the clock positions of the LRA and RRA. A statistically significant change within the FA group was seen for the SMA tortuosity index and the angles of the LRA and RRA. Statistically significant differences between groups were seen in the aortic diameter changes at the SMA and renal arteries, the change of the LRA and RRA clock position, and in the SMA angle distal of the target vessel stent.

**Table 5.** Anatomical Change Before and After Implantation Within and Between Groups.^a^

Variable	Zenith Fenestrated	Fenestrated Anaconda	p
Aortic diameter at the SMA, mm	−3 (−4 to −1) p<0.001 [110]	1.0 (−1 to 2) p=0.68 [35]	<0.001
Aortic diameter at the RA, mm	−2 (−4 to −1) p<0.001 [110]	0.0 (−2 to 2) p=0.87 [35]	<0.001
CA tortuosity index	−0.02 (−0.07 to 0.28) p=0.29 [10]	0.01 (–0.03 to 0.13) p=0.72 [4^b^]	0.36
CA clock position, deg	−4 (−5 to 3) p=0.29 [10]	−4 (−14 to 4) p=0.47 [4^b^]	0.84
Angle of CA, deg	−14 (−17 to −7) p=0.012 [10]	−6 (−19 to 1) p=0.29 [4^b^]	0.40
SMA tortuosity index	0 (−0.01 to 0.02) p=0.19 [47]	0.02 (0 to 0.05) p=0.04 [18]	0.08
SMA clock position, deg	−1 (−6 to 6) p=0.92 [47]	−1 (−10 to 5) p=0.70 [18]	0.56
Angle of the SMA, deg	−4 (−11 to 4) p=0.02 [47]	−4 (−11 to 9) p=0.46 [18]	0.61
LRA tortuosity index	0 (−0.04 to 0.04) p=0.59 [105]	0.01 (−0.07 to 0.04) p=0.58 [32]	0.84
LRA clock position, deg	−4±11 p<0.001 [105]	2±15 p=0.67 [32]	0.019
Angle of the LRA, deg	−9 ±16 p<0.001 [105]	−13±19 p=0.001 [32]	0.28
RRA tortuosity index	−0.01 (−0.04 to 0.02) p=0.13 [98]	−0.02 (−0.05 to 0.03) p=0.33 [34]	0.70
RRA clock position, deg	5±12 p<0.001 [98]	1±14 p=0.774 [34]	0.06
Angle of the RRA, deg	−10±14 p<0.001 [98]	−12±14 p<0.001 [34]	0.48
CA angle stent^c^	157 (141 to 167) [10]	148 (133 to 150) [4]	0.14
SMA angle stent^c^	163 (158 to 168) [47]	169 (167 to 173) [18]	0.003
LRA angle stent^c^	150±17 [104]	156±14 [32]	0.09
RRA angle stent^c^	149±12 [98]	150±15 [34]	0.58

Abbreviations: CA, celiac artery; LRA, left renal artery; RA, renal arteries; RRA, right renal artery; SMA, superior mesenteric artery.

a Continuous data are presented as the mean ± standard deviation or median (interquartile range Q1, Q3) [sample size] with p values for the comparison of preoperative vs postoperative measurements.

b In 1 case a celiac artery could not be stented; it was intentionally covered with a cuff.

c Stent angles are measurable only on the postoperative scans.

No difference between BE covered and SE bare metal or combined stents was seen in the anatomical change of the LRA and RRA tortuosity indices (p=0.295 and p=0.734, respectively), clock position (p=0.457 and p=0.060, respectively), or angle relative to the aortic CLL (p=0.774 and p=0.882, respectively). Only covered stents were used in the CA, and a combination of stents was used in only 1 SMA, so no comparison could be tested. No difference was observed between stents in the target vessel angle distal of the stent for the LRA (p=0.396) or RRA (p=0.863).

## Discussion

The implantation of a fenestrated endograft altered the anatomy of the proximal abdominal aorta and its visceral branches, without large differences between the 2 endograft models. In both groups, anatomical changes were observed for angles in the renal arteries but not in the mesenteric arteries. Endograft planning was done relative to the mesenteric arteries, potentially leading to a mismatch in measurements of the renal arteries. Alternatively, the position of the mesenteric arteries is more rigidly fixed to the surrounding tissues, and as a consequence they may be less influenced by stenting. Furthermore, the renal arteries are more often involved in the aneurysm than the mesenteric arteries, explaining their higher mobility. The sample size of stented renal arteries was higher than mesenteric arteries, and results of stented mesenteric arteries could therefore be false negative.

A difference between groups was seen in the change of the clock positions of the renal arteries. In the ZF group, renal artery clock position moved anteriorly, while this was not seen in the FA group. During placement of the ZF, the endograft is partly deployed, the target vessels cannulated, the diameter-reducing ties were released, and finally the target vessels stented; as a consequence, after release of the diameter-reducing ties, the stented arteries can be pushed anteriorly. Furthermore, the main part of the FA is unrestricted by circular stents, and the aortic blood pressure can push the graft to the aortic wall. Alternatively, the circular Z-stents of the ZF prevent expansion, resulting in aortic size alteration. Consequent to the expansion of the main part in the FA, the distal part of the endograft could have moved upward, which might lead to folding of the graft material.

The angle distal to the stent in the SMA and a perpendicular movement of the SMA relative to the aortic CLL were more pronounced in the ZF group. The absence of a SMA angle change in the FA group was at the level of the FA unrestricted by circular stents, which allowed the fenestration to move after stenting. One would expect to see a change in other target vessels of the ZF group too, but this was seen only to a limited extent for the LRA in our study.

The observation that the tortuosity index of target vessels did not differ between groups may seem logical because similar BE covered stents were used to bridge the fenestrations in most cases of both groups. Nevertheless, the docking of these stents in the fenestrations is more rigid in the ZF group due to their position relative to the struts of the Z-stent. This may well be the reason that the clock position of the renal arteries and the angles of the SMA and CA changed significantly in the ZF group and not in the FA group. A main body that is unrestricted by struts has greater adaptation to native anatomy and may therefore have less risk of strain on the stents and consequently less chance of stent fractures. Nevertheless, both devices caused an equal straightening of the renal arteries, and change in the SMA tortuosity index was observed only in the FA group, indicating that the difference between the endografts in terms of the conformational change of the target vessels is probably limited.

The conformability of an endograft is an important factor for outcome prediction after EVAR, especially in those with more severe aortic and iliac angulations and iliac tortuosity indices.^14–17^ As shown in this study, there are anatomical changes of the stented visceral arteries after implantation of a fenestrated endograft. An increased risk of renal and neurological injury has been described after fEVAR, but the exact relation to endograft conformability is yet unknown.^25^ One of the considerations in choosing an endograft should include the angles and tortuosity indices of the aorta, the iliac arteries, and visceral arteries, as well as the ability of the endograft to conform to the patient’s anatomy. After implantation a comparative analysis should be done between the preoperative and postoperative CTA to predict complications related to endograft conformability. Unfortunately, due to small group and event numbers in our study, a reliable regression analysis could not be performed to explore any relationship between conformability and the complications and reinterventions. Furthermore, not all clinical follow-up data were available. Moreover, the rather large number of cases excluded because the CTAs did not meet the slice thickness criterion involved ZF procedures between 2002 and 2007. Consequently, selection bias cannot be ruled out. Subsequent studies aimed at clinical results related to anatomical changes after endograft implantation should be performed to find the influence of geometrical changes on long-term outcome.

Treatment with the fenestrated endograft for complex AAA was primarily done in patients unfit for open surgery.^26^ Over time fenestrated EVAR was also chosen for patients with fewer comorbidities. The time gap in our cohort between introduction of the ZF and the FA was nearly 10 years and may explain the differences in comorbidities. By the time the FA was introduced, significant experience with complex endovascular interventions had been gained, which could explain the lower procedural blood loss in the FA patients.

Though there were no statistically significant differences in baseline characteristics between the groups, there were relatively more cases with a juxtarenal aneurysm in the ZF group. The difference might be a result of variations in practice. The earlier introduction of the ZF might result in a broader experience that favored fEVAR over open repair. The same is true for a slightly higher number of fenestrations in the FA group. The potential flexibility of the FA might have resulted in choosing fEVAR over open repair. No difference was seen between cases with previous surgery for AAA. It should be kept in mind that the implanted endograft might have no influence on the aortic diameter, but in these cases the stented target vessels were considered native.

No difference was seen in anatomical change between BE and SE stents for target vessels, but it might be a confounder for anatomical change. The same might be the case for different preoperative anatomy, patient selection, and the endograft instructions for use. These confounders should be taken into account when interpreting the results.

Customizing the endograft is time-consuming and led to a delay of a few months between baseline CTA and treatment. The exact time between industry contact and final approval was not known, but possibly this took more time for the ZF, especially in the early period, potentially explaining the differences in the intervals between the CTA and treatment for the groups. With regard to the differences between both groups in timing of the first follow-up, one center routinely performed the first postoperative CTA at around 4 weeks postimplantation, while the other center (having implanted the FA only) performed the first postoperative CTA before patient discharge.

The ICC was high for nearly all variables, both between 2 repeated measurements by the same observer and between the 2 independent observers. The measurements for different variables were performed in the same way. All pre- and postoperative aortic diameters, target vessel tortuosity indices, target vessel clock positions, and target vessel angles relative to the aortic CLL were combined. It would be ideal to analyze variables separately, but the sample sizes were too small to have a reliable agreement for individual variables. Consensus in anatomical measurement techniques, making them reproducible, might eventually help prevent errors in measurements and, consequently, clinical complications.^27^

Pressure changes during the cardiac cycle can influence anatomical configurations. In this study, the CTA did not account for the cardiac cycle. Dynamic CTA allows scanning at specific moments during the cardiac cycle.^28^ Measuring these changes is too complex and too labor intensive. Computer algorithms might help in future research to show continuing movement of the endograft.^29^

The influence of small anatomical changes on outcome is still unclear, and the clinical consequences and cutoff values for clinical relevance need to be examined. A large prospective randomized trial with both endografts would help understand which patient benefits the most from which fenestrated endograft, but it is unrealistic due to the large series needed to show very small changes in anatomy and relations between different variables. Until the clinical relevance of these changes has been shown, our study shows good conformability of both designs.

## Conclusion

The implantation of a custom-made fenestrated endograft for complex AAAs seems to alter vascular anatomy, but there is no difference between current commercially made endografts for the target vessel tortuosity index. This study suggests that conformability may be different for the ZF and the FA. Further studies are necessary to elucidate the relation to clinical outcome.
